# Valorization of Potato Peel Waste into Bioactive Compounds and Sustainable Bioplastics Production Through a Novel Biorefinery Approach

**DOI:** 10.3390/polym17243339

**Published:** 2025-12-18

**Authors:** Rijuta Ganesh Saratale, Ganesh Dattatraya Saratale, Han Seung Shin

**Affiliations:** 1 Research Institute of Integrative Life Sciences, Dongguk University-Seoul, Ilsandong-gu, Goyang-si 10326, Gyeonggido, Republic of Korea; griju22@gmail.com; 2 Department of Food Science and Biotechnology, Dongguk University-Seoul, Ilsandong-gu, Goyang-si 10326, Gyeonggido, Republic of Korea; ganeshfbtdongguk17@gmail.com

**Keywords:** potato peel waste, polyhydroxyalkanoates, *R. eutropha* ATCC17699, acid pretreatment, antidiabetic activity

## Abstract

This study deals with the successful exploitation of easily available and renewable potato peel waste (PPW) as an excellent feedstock in the production of PHA using *Ralstonia eutropha*. The process entailed the extraction of bioactive components from PPW by use of solvent-based procedures and screening of their antioxidant and antidiabetic activity. The extracted PPW biomass was subject to acid hydrolysis using different concentrations of sulfuric acid for hydrolysis and solubilization of sugar components. The obtained liquid (acid) hydrolysates were initially assessed to biosynthesize PHA. Activated charcoal-based detoxification of acid hydrolysates was observed to be more efficient in promoting bacterial growth and accumulation of PHA. Acid-pretreated PPW biomass was further enzymatically hydrolysed to accomplish full saccharification and used to produce PHA. The effects of provision of nutrients and employing stress state conditions were assessed to improve bacterial growth and PHA accumulation. In both hydrolysates under optimal conditions, *R. eutropha* demonstrated the highest biomass productivity of 7.41 g/L and 7.75 g/L, PHA accumulation of 66% and 67% and PHA yield of 4.85 g/L and 5.19 g/L, respectively. XRD, FT-IR, TGA and DSC analysis of produced PHA were studied. The results showed that the produced PHA displayed similar physicochemical and thermal properties to commercially available PHB. Overall, this work illustrates the possibilities of abundantly available PPW, which can be transformed into bioactive compounds and high-value bioplastics via a coupled bioprocess. This approach can develop process economics and sustainability within a cyclic biorefinery system and serve further industry applications.

## 1. Introduction

The United Nations projects that by 2050, the world’s population is estimated to rise to about 9.8 billion people [1]. Economic growth and changes in diets increase consumption of food, which in turn increases global food waste. It is estimated that, every year, about 1.3 billion tons of food are discarded or misplaced [2,3]. Transforming the wastage of food into value-augmented products generates economic profits, along with supporting the sustainability objectives as per the Environmental Protection Agency (EPA) and the Food and Drug Administration (FDA) [4,5].

The potato (*Solanum tuberosum*) belongs to the *Solanaceae* family and is the third most produced and consumed food crop in the world after rice and wheat [6,7]. Potatoes are produced at a rate of approximately 370 million tons annually on a global scale [2]. A large portion of this is industrially and domestically processed, with peeling the outer skin of the tuber commonly being the initial stage. During this process, huge amounts of potato peel waste (PPW) are obtained. Depending on the peeling method used, the generation of PPW accounts for about 15 to 40% of the tuber’s original weight and volume [1]. Landfilling, a non-sustainable approach, is traditionally used for the management of the PPW biomass; however, it contributes to environmental pollution [8,9].

The sustained supply, high nutritional value and relatively homogenous composition consisting of starch, polyphenols, alkaloids, non-starch polysaccharide, lignin and proteins with minor lipid content make PPW a promising consideration in terms of biorefinery applications [7,10]. In spite of these benefits, direct utilization of PPW as a carbon source in microbial fermentation can be impeded due to certain inhibitory compounds, such as glycoalkaloids and polyphenols, that may negatively affect microbial growth [11,12]. Hence, it is significant to eliminate these bioactive inhibitors prior to transforming PPW into high-value bioproducts. However, these bioactive compounds, including phenolic acid, flavonoids and glycoalkaloids, ensure PPW is a resource that can be used to develop functional food products applicable in human as well as animal nutrition. Diabetes mellitus (DM) is a metabolic disorder affecting hundreds of millions of people worldwide, and it is one of the critical health concerns in the world nowadays. High levels of reactive oxygen species (ROS) lead to oxidative stress, which is one of the key contributors to the onset of aging-associated diseases such as cancer and cardiovascular disease. It has been recently demonstrated that polyphenols are powerful antioxidants able to inhibit digestive enzymes that take part in the metabolism of carbohydrates. Thus, the extracted bioactive compounds of PPW can be used as a powerful antioxidant and to treat hyperglycaemia.

Moreover, a substantial fraction of PPW is lignocellulosic biomass with a recalcitrant and complex structure. Such structural recalcitrance needs to be dealt with to attain efficient enzymatic hydrolysis, which can be obtained through appropriate pretreatment processes [13,14]. Acid hydrolysis emerged as one effective pretreatment technique owing to its numerous benefits. Its application is comparatively easy for substrates containing high starch content, as it offers a rapid reaction rate, requires simple cheap acid catalysts and can be used in relatively mild temperature conditions [15,16].

Fossil fuel-generated plastic materials are broadly utilized in contemporary society because of their great durability and multi-functionality. The use of plastic materials, however, has resulted in major environmental issues because of its accumulation in both terrestrial and marine systems [17]. Polyhydroxyalkanoates (PHAs) have generated intense research attention as they are biodegradable and biocompatible in nature with structural diversity. PHAs are used in various industries such as biomedicine, electronics, construction, automotive, packaging and agriculture. The PHA market in the world is projected to comprise approximately 14% of the total bioplastics market [18,19,20]. PHAs are mainly aliphatic polyesters synthesized by different microorganisms in the cytoplasm, where they accumulate in the form of insoluble carbon and energy storage materials. Biopolymers tend to occur when carbon is abundant and nutrient conditions are poor (especially nitrogen or phosphorus) [21]. Although PHAs show promising characteristics, they are still far costlier than traditional petrochemical plastics, posing a critical impediment to the adoption of PHAs market [22]. Almost half of the total production cost of PHA is recognized as the cost of the carbon source, which makes it the leading bottleneck in microbial production of PHA with the current system. One of the most studied PHA-producing bacteria is *Ralstonia eutropha* (*Cupriavidus necator*), which can utilize a broad variety of inexpensive, renewable carbon resources to biosynthesize PHAs [23]. *R. eutropha* production of PHAs primarily follows the β-oxidation pathway, with the help of important enzymes acetoacetyl-CoA synthetase and PHB synthase for the transformation of acetyl-CoA to polyhydroxybutyrate (PHB) [24].

**Figure 1:**
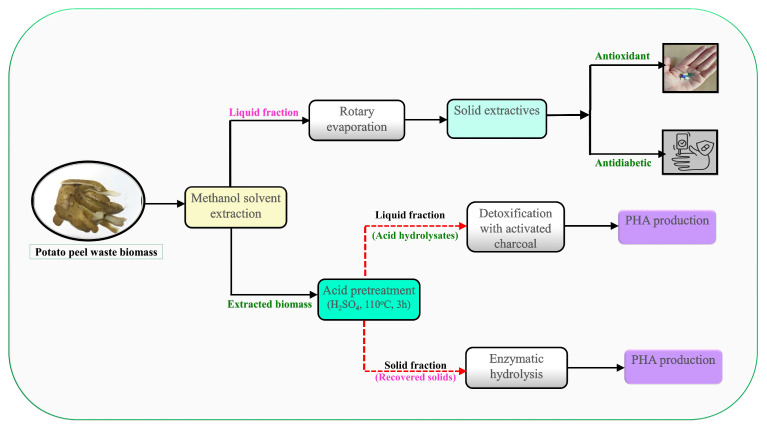
Schematic presentation of the proposed research study.

This study reveals the significance of potato peel residues as a renewable resource to produce bioactive compounds and PHAs in a sustainable manner, helping the expansion of a circular bioeconomy and open a range of opportunities to industrial use. The use of PPW seems to be an integrated biorefinery strategy in the context of sustainable PHA production and efficient waste management. This method provides economic, environmental and industrial advantages. The procedure started with extraction of bioactive molecules in PPW using a solvent methodology. Then, dilute acid pretreatment was adopted to solubilize sugar components by deconstructing the compact structure of PPW biomass. Enzymatic saccharification of the acid-pretreated PPW was further performed to maximize the release of fermentable sugars. The acid hydrolysates and enzymatic hydrolysates of PPW were used as carbon sources for PHA production by *R. eutropha*. The optimal experimental conditions, nutrient supplementation and stress conditions were examined to maximize PHA production. Lastly, the physico-chemical properties of the produced PHAs were determined using standard analytical methods. The schematic representation of the proposed research study is presented in Figure 1.

## 2. Materials and Methods

### 2.1. Collection, PPW Processing and Microbial Strains

In this study, potato peel waste was utilized as the raw material and was bought from vendors who were proficient and certified in the Goyang-si market of South Korea. After acquiring it, the biomass was completely cleansed to eliminate impurities and then dried at a 40 °C temperature until a stable weight was achieved. After drying, the material was milled to decrease its particle size of 0.5 mm. The processed PPW biomass was then put in sealed plastic bags in a refrigerator to preserve its integrity until its use. During the experiment, DDW refers double-distilled water (Millipore, Billerica, MA, USA), which was utilized in the preparation of solutions. Moreover, all the chemicals employed during the acid pretreatment, synthesis of PHA, extraction and further analysis had the highest purity and analytical quality. The *Ralstonia eutropha* ATCC 17699 bacterial strain was purchased from the American Type Culture Collection (ATCC, Manassas, VA, USA). This bacterium was sub-cultured using a broth of Tryptic Soy with no dextrose (TSB; Becton Dickinson, Franklin Lakes, NJ, USA) and incubated at 4 °C under ordinary conditions. Routine subculturing was performed after every month to maintain the viability of the culture.

### 2.2. Phytochemical Extraction of PPW and Biogenic Potential

The maceration of the powdered form of PPW was performed in 80% methanol and water mixture at 1:10 (*w*/*v*). This process was performed at 65 °C for 3 h by keeping the reaction mixture in a shaking incubator (200 rpm) under dark condition. Three repetitions of this process were executed to make sure that the phytochemicals were completely extracted. The filtration was performed using Whatman No. 91 filter paper after every extraction. The filtered extracts were mixed and concentrated through a biconical rotary vacuum evaporator to obtain a dry extract. To identify the yield, the weight of the obtained powder was measured, and it was kept at −20 °C to be analyzed later. Qualitative determination of phytochemical constituents of PPW extractives such as alkaloids, tannins, terpenes, saponins, flavonoid, steroids, glycoside, phenol and anthraquinones was performed based on pre-established protocols [25]. The total phenolic content (TPC) of the extract was evaluated by following the Folin–Ciocalteu method [26]. The content of flavonoids was evaluated as per the procedure described by Kumaran and Joel [27], with rutin being the reference compound. To do this, 1 mL of the PPW extract (10 mg/mL in methanol) and 1 mL of aluminum trichloride were combined in 20 mg/mL of ethanol, followed by the addition of a drop of acetic acid (undiluted, 97–99% purity). After 40 min, the mixture was diluted with 25 mL of ethanol, and the absorbance was recorded at 415 nm.

#### Antioxidant and Antidiabetic Potential of PPW Solvent Extractives

Determination of the antioxidant activity of the extracts of PPW was tested according to their ability to scavenge free radicals, namely DPPH and ABTS assays, and the test was performed following the previous reported protocols [28]. The antidiabetic activity of the extracts was evaluated by investigating their inhibitory activities on the major enzymes hydrolysing carbohydrates, i.e., α-amylase and α-glucosidase using acarbose as a reference. The enzyme activity assays were conducted as per the standard procedures [29] and three replicate values were recorded for each assay. The antioxidant and antidiabetic activity of the PPW extracts was measured using the mean values of these replicates. IC_50_ values state the concentration of PPW extractives needed to attain 50% inhibition of antioxidant and antidiabetic enzyme activity, which were determined based on the formulas reported earlier [28,29].

### 2.3. Acid Treatment of the Extracted PPW Biomass

The extractive-free PPW biomass was reclaimed and air dried in order to remove any solvent residue. Dried biomass was hydrolyzed with sulphuric acid (H_2_SO_4_) with concentrations of 0.5, 1.0, 1.5 and 2.0% (*v*/*v*). The biomass of PPW was suspended in the acid solution with the proportion of solid to liquid being 1:10 (*w*/*v*) in each treatment. The solution was then incubated in a water bath at 100 °C for approximately 3 h. Essentially to compare the effect of the preceding step of extraction, unextracted PPW biomass was also subjected to 1% (*v*/*v*) of H_2_SO_4_ at 100 °C and 3 h incubation under the same solid-to-liquid ratio. Furthermore, physicochemical pretreatment was carried out. In this method, PPW biomass mixed with 1% (*v*/*v*) solution of H_2_SO_4_ was autoclaved at 121 °C for 20 min. At the end of every pretreatment, reaction mixtures were centrifuged at 4000 rpm for 20 min to isolate liquid (acid) hydrolysate and solid residue (Labogene, 1736R, Lillerod, Denmark). The following formula was used to calculate the solid recovery of the pretreatment process.

(1) $$Solid\;recovery=\frac{Recovered\;solids\;remaining\;after\;pretreatment\;[g]}{Weight\;of\;the\;sample\;[g]}\times 100$$

Meanwhile, the acid-treated solid biomass was thoroughly washed using distilled water to reach a neutral pH. The neutralized product was dried in a hot air oven (60 °C) until constant weight was achieved and further used for enzymatic saccharification experiments.

#### Detoxification of PPW Acid Hydrolysates

After acid pretreatment the liquid fraction was collected and subsequently detoxified with the activated charcoal in an effort to eliminate fermentation inhibitors such as furfural and hydroxymethylfurfural (HMF). To carry out detoxification, 25 g of activated charcoal was added per liter of acid hydrolysates. This mixture was stirred at 150 rpm at 45 °C for 60 min, after which centrifugation at 1100× *g* for 15 min was performed to separate the charcoal as described by Mussatto et al. [30]. Reducing sugar and inhibitory compound levels were determined prior to and after the detoxification process to determine the effectiveness of the process.

### 2.4. Enzymatic Degradation of Acid-Pretreated PPW with the Crude Enzyme Complex

The obtained acid-pretreated PPW biomass was enzymatically hydrolyzed with a crude enzyme cocktail produced by *Streptomyces* sp. MDS. The enzyme was produced and extracted from solid-state fermented wheat straw biomass under optimized growth conditions [31]. The total cellulase activity (measured in filter paper units, FPU) of the crude enzyme was determined through standard protocol of IUPAC in which Whatman filter paper was employed as substrate [32]. Enzyme activity was measured in units in which the unit denotes the quantity of enzyme catalyzing the release of 1 mmol of reducing sugars/min under the assay conditions. The untreated and acid-pretreated PPW biomasses of 2.0% (*w*/*v*) concentration were used in enzymatic hydrolysis experiments. Hydrolysis was carried out with 20 mL of citrate buffer (50 mM, pH 5.0) and sodium azide of 0.005% (*w*/*v*) was augmented to exclude the possibility of microbial contamination. The crude enzyme complex was included in a dosage equal to 30 FPU/gram of PPW. The performance of the enzyme complex was assessed by changing the PPW concentration between 5 and 25 g/L, in which the enzyme dose was kept constant (30 FPU/g). In the case of the other set, enzyme dosage varied in concentration between 10 and 50 FPU/g, and PPW concentration was maintained at 10 g/L. Enzyme hydrolysis was evaluated by the concentration of the reducing sugars that were liberated in the course of hydrolysis to check its efficiency. The hydrolysis yield and glucose yield were calculated using the formulas reported earlier [31].

### 2.5. PHA Production Using Acid Hydrolysates and Enzymatic Hydrolysates of PPW by R. eutropha

PHA production using acid hydrolysates and enzymatic hydrolysates of PPW as carbon sources was tested using *R. eutropha* [23]. Firstly, the growth and PHA accumulation of *R. eutropha* were assessed through the aid of acid hydrolysates, which were prepared before and after detoxification with activated charcoal. Simultaneously, enzymatic hydrolysates of acid-pretreated biomass of PPW were also tested for PHA production. The PHA fermentation medium was prepared by adding a defined mixture of mineral salt solution with the following constituents (g/L): NaH_2_PO_4_, 3.6; K_2_SO_4_, 3.486; Na_2_HPO_4_, 2.84; NaOH, 0.4; MgSO_4_·7H_2_O, 0.39; (NH_4_)_2_SO_4_, 0.1; yeast extract, 0.2; CaCl_2_, 0.062; MnSO_4_·H_2_O, 0.024; CuSO_4_·5H_2_O, 0.005; FeSO_4_·7H_2_O, 0.15; and ZnSO_4_·7H_2_O, 0.024, pH 7.0. In the culture medium, acid hydrolysates and enzymatic hydrolysates of PPW were combined to attain an around 20 g/L initial sugar concentration. In the case of PHA production, inoculation of 5 mL (*v*/*v*) of actively growing *R. eutropha* culture in 100 mL of the production medium in 250 mL Erlenmeyer flasks was carried out. The fermentation was carried out under batch conditions and cultivation temperature was kept at 30 °C, with pH 7.0 and fermentation, and was agitated at 200 rpm with a total fermentation time of 48 h. These parameters were applied consistently in all experiments on PHA production.

### 2.6. Nutrient Supplementation Effects on PHA Accumulation

The effects of nutrient supplementation into the fermentation medium were investigated to enhance the bacterial cell growth and PHA production. Effects of supplementation of the nitrogen sources corn steep liquor (CSL) and peptone at 1% (*v*/*v*), along with effects of each of the individual volatile fatty acids (VFAs), namely, butyrate, propionate and acetate at 1% (*v*/*v*), were tested. Further, the effect of external osmotic stress was evaluated by adding NaCl at concentration levels of 5 g/L, 10 g/L and 15 g/L (*w*/*v*). In order to ensure reliability and reproducibility, all experiments were performed three times to determine average values to be considered.

The cell growth of *R. eutropha* and PHA accumulation parameters were calculated by employing the following formulas:

(2) $$PHA\;accumulation\;(\%)=\frac{Extracted\;quantity\;of\;PHA\;(g/L)}{Dry\;cell\;Weight\;(g/L)}\;\times 100$$

(3) $$PHA\;yield\;coefficient\;owing\;to\;cell\;biomass\;(Yp/b)=\frac{PHA\;final\;quantity\;(g/L)}{Dry\;cell\;Weight\;(g/L)}$$

(4) $$HA\;yield\;coefficient\;owing\;to\;substrate\;consumption\;(Yp/s)=\frac{PHA\;final\;quantity\;(g/L)}{PPW\;hydrolysates\;utilized\;(g/L)}$$

### 2.7. Extraction and Purification of PHA

The bacterial biomass was acquired following the fermentation process by centrifugation (10,000× *g*) over 10 min at 4 °C (Labogene, 1736R, Lillerød, Denmark). The obtained cell pellet was subjected to extensive washing to eliminate impurities and then freeze dried to achieve a stable and dry cell mass. In the case of PHA extraction, the lyophilized biomass was initially subjected to a mixture of chloroform and sodium hypochlorite solution to lyse the cells. PHA polymer was subsequently precipitated in the presence of 80% methanol and vacuum filtrated [23]. The resulting PHA polymer in a white form was washed two more times with a mixture of methanol and chloroform and further dried at 60 °C in a hot air oven for a duration of 48 h. The pure PHA was then subject to further analytical characterization.

### 2.8. Analytical Methods

The contents of cellulose, hemicellulose and lignin of PPW were determined by the Goering and Van Soest methods [33]. The presence of fermentable sugars (mainly glucose, xylose and arabinose) and other soluble metabolites in PPW acid hydrolysates was quantified utilizing HPLC (Agilent model 1200, Palo Alto, CA, USA). The starch content was determined by the Megazyme Total Starch Assay Kit (Amyglucidase/a-amylase method, K-TSTA-50A/K-TSTA-100A, Wicklow, Ireland) according to the AOAC Method 996.11 and AACC Method 76-13.01. The nitrogen content of the biomass was determined using N elemental analyzer (Thermo Finnigan EA 1112, Waltham, MA, USA). The concentration of the protein was then determined by a nitrogen-to-protein conversion factor of 6.25 g of protein/g of nitrogen. Reducing sugars (RS) was performed with the aid of the dinitrosalicylic acid (DNS) procedure [34]. Scanning electron microscopy (SEM) was used to monitor the surface morphology of untreated and acid-treated PPW biomasses. A JEOL JSM-6360A microscope (Tokyo, Japan) was used to acquire SEM images at an operating voltage of 20 kV. The composition of functional groups of the produced PHA from untreated and acid-pretreated PPW biomasses was determined using an FTIR spectrometer (Cary 630 FTIR, Agilent, Santa Clara, CA, USA) with a range of 4000–400 cm^−1^ and resolution of 4 cm^−1^. The PHA was analyzed using X-ray diffraction (XRD) by maintaining standard conditions using an X-ray diffractometer (D2 Phaser (Bruker, Berlin, Germany)). The examination was conducted at room temperature. Scan tests were run in the 2θ range of 10° to 50° at a rate of 1°/min. The thermal decomposition behavior of the produced PHA was assessed through thermogravimetric analysis (TGA) through the use of a Hi-Res TGA 2950 thermogravimetric analysis instrument (TA instruments, New Castle, DE, USA). Approximately 10 mg of the sample of PHA was heated between 20 °C and 500 °C in increments of 20 °C/min under a nitrogen atmosphere. The glass transition temperature (T_g_) and melting temperature (T_m_) of the PHA samples were measured using differential scanning calorimetry (DSC). About 2 mg of synthesized PHA sample was hermetically sealed and subjected to thermal analysis in a DSC 2920 system (TA Instruments, New Castle, DE, USA). It was initiated at the equilibrium of −50 °C to 250 °C, keeping the heating rate at 10 °C/min and the cooling rate at 5 °C/min. The elaborate details concerning the conditions of the sample preparation and the methods of TGA and DSC analysis were chosen according to the standard protocols described [23].

### 2.9. Statistical Analysis

A one-way analysis of variance (ANOVA) was employed to determine the obtained data. After completion of the ANOVA, Tukey’s HSD test was conducted to calculate the specific differences in the means statistically, through application of GraphPad InStat software version 3.06 (GraphPad Software Inc., San Diego, CA, USA). All tests were applied at a significance level of *p* = 0.05, which was considered to test the differences in the means.

## 3. Results and Discussion

### 3.1. Methanol Extraction and Chemical Analysis of Bioactive Compounds of PPW

The extraction of valuable phytonutrients, especially polyphenolic compounds, from potato peels represents a sustainable approach. The approach not only has health value but also enhances the value of industrial by-products that are generated during potato processing. The chemical composition of methanolic extracts obtained in PPW has shown the presence of a wide spectrum of bioactive constituents including terpenoids, alkaloids, tannins, phenols, flavonoids and glycosides. The TPC of the methanolic extract was determined as 2.61 ± 0.45 mg GAE/g DW (Table 1). This value aligns with other reported values; for instance, Mohadly et al. [35] recorded 2.91 mg GAE/g DW in methanol-extracted potato peels, superior to the TPC of sugar beet pulp of about 1.79 mg GAE/g DW and sesame cake extracts, which showed 0.81 mg GAE/g DW. However, the concentration of the TPC in acidified ethanol extracts of PPW showed a higher TPC of about 14.03 mg GAE/g DW [36]. Another important group of polyphenolic compounds present in PPW are referred to as flavonoids, which are characterized by their multiple biochemical activities and free radical scavenging properties. In this regard, the total flavonoid content (TFC) of PPW extracts is also intended (Table 1). The obtained TFC value, 0.806 ± 0.05 mg QE/g DW, correlates with previous research with results such as a methanol-extracted PPW of 0.96 mg QE/g DW [35] and was lower than for the acidified ethanol extract of PPW, at 3.310 mg QE/g DW [36]. The differences in both the values of TPC and TFC between various studies can be attributed primarily to differences in cultivars of potatoes and the methods used in extraction, as highlighted by Sarangi et al. [4].

### 3.2. Antioxidant and Antidiabetic Activity of Extracted Bioactive Compounds of PPW

**Figure 2:**
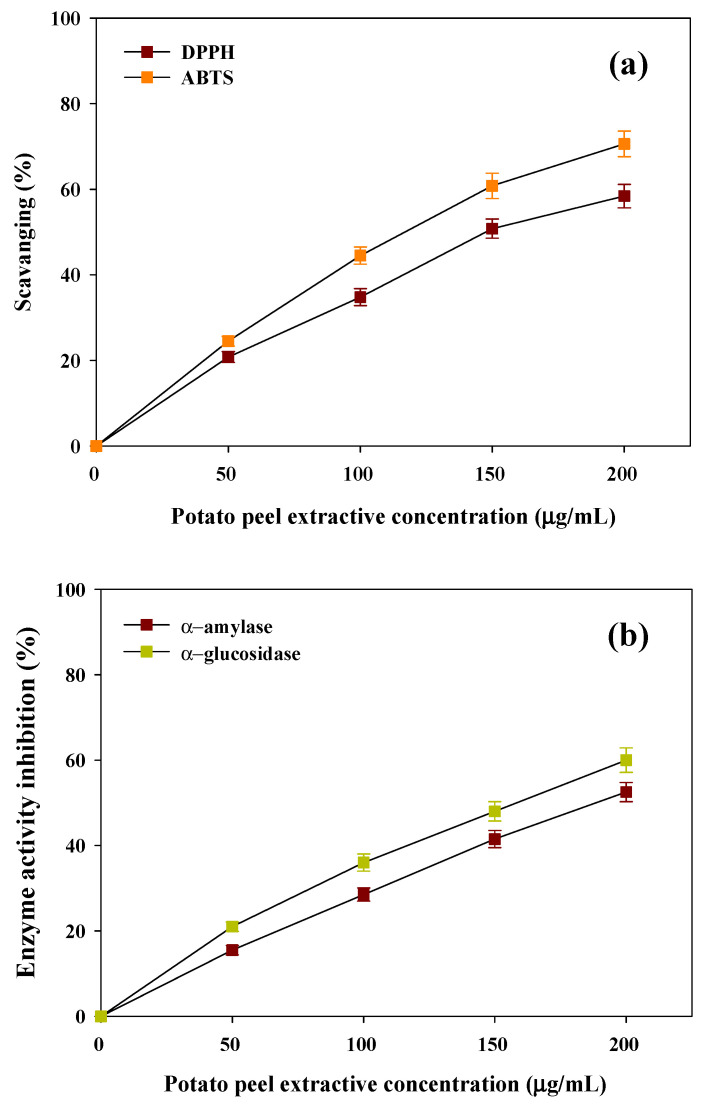
Biogenic potential of extractives of PPW’s (**a**) antioxidant potential against DPPH and ABTS and (**b**) antidiabetic potential against α-amylase and α-glucosidase.

As per the International Diabetes Federation (IDF), there are around 537 million people worldwide living with diabetes according to the newest figures [37,38]. A modern lifestyle is closely linked to the rising trend of DM, which is becoming a major concern in the population. Hyperglycemia leads to the excessive formation of reactive oxygen species (ROS), which stimulates oxidative destruction to important cellular components including DNA, proteins and lipids, which are essential biomolecules. In light of this, antioxidants are widely being investigated for therapeutic effects in alleviating the oxidative stress and its associated diabetes complications [38,39]. The antioxidant activities of PPW methanolic extracts were tested based on DPPH and ABTS radical scavenging activities. The findings showed that PPW extract produced a concentration-dependent free radical scavenging antioxidant activity against DPPH and ABTS. The powerful antioxidant capability of PPW extract was confirmed by the lower IC_50_ of DPPH (150 ± 4.15 µg/mL) and ABTS (115 ± 3.32 µg/mL) radicals, respectively (Figure 2a). The substantial antioxidant and cytotoxic effects are mostly attributed to the level of phenolics and the redox properties of the extracts [40,41].

There has been emerging evidence that shows that a strong negative relationship exists between total polyphenol content and the glycemic response [42]. The antidiabetic effects of PPW extract were examined in terms of the ability to hinder the activities of the carbohydrate hydrolysing enzymes α-amylase and α-glucosidase. Figure 2b reveals that the inhibitory activity of PPW extract regarding enzymes, which exhibited a positive correlation with the concentration of the extracts. The substantial anti-diabetic properties of PPW extract are indicated by the moderate values of IC_50_ against α-amylase and α-glucosidase (183 ± 4.45 µg/mL and 156 ± 4.85 µg/mL, respectively). The presence of inhibitory activity of these essential enzymes shows that they could be useful in management of hyperglycemia (Figure 2b). The above findings suggest that extraction and recovery of such bioactive compounds not only helps in waste management but also contributes a significant source of economic profitability to the food industry.

### 3.3. Effects of Acid Pretreatment on the Chemical Composition of PPW

The chemical composition of potato peel waste (PPW) biomass showed a content in (g/100 g of dry PPW) are presented in Table 2. PPW consists of polysaccharides, primarily starch and lignocellulose (~66.6 g/100 g of dry PPW). This composition further revealed that PPW could serve as a source of industrially viable products like biofuels, biochemicals and polyhydroxyalkanoates (PHAs).

Chemical pretreatment has been widely used for the hydrolysis of various lignocellulosic biomasses. The efficiency of chemical pretreatment relies on the type of chemical used, its concentration, operation conditions and incubation time. Dilute acid pretreatment is a significant chemical pretreatment method for its simplicity; it is cost-effective and has a higher efficiency in lignin removal by disrupting the lignocellulosic composite material. In this process, H^+^ concentration directly affects the hydrolysis of biomass and cleaves the heterocyclic ester bonds between sugar monomers in the polysaccharide chains of cellulose and hemicellulose.

Optimization of pretreatment parameters was studied by using a 1% H_2_SO_4_ concentration, where significant hydrolysis of PPW biomass and delignification were observed at a pretreatment thermal temperature of 100 °C and treatment time of 3 h. All pretreatment studies with different concentrations of sulfuric acid were subjected to these optimized conditions. Mild acid treatments with high temperatures and prolonged durations have been shown to increase the solubility of sugar fractions and reduce inhibitory compound formation [43,44]. This supports our results on efficient sugar extraction with minimized inhibitory compound formation.

Dilute sulfuric acid pretreatment for the hydrolysis of the lignocellulosic biomass resulted in solubilization of starch and hemicellulose components and effective delignification of PPW. After performing the acid pretreatment, a considerable amount of PPW (21.5–58.0%) was solubilized into the liquid. After increasing the acid concentration solubilization of starch components from 52.5 to 88.8%, lignin removal rising from 30.8 to 88.3% and hemicellulose from 35.6 to 70.2% were observed (Table 3). The decrease in solid biomass after pretreatment is primarily because of the solubilization of starch, hemicellulose and lignin components into the surrounding aqueous medium [45].

Similarly, acid pretreatment with 0.1% (*w*/*v*) H_2_SO_4_ at 121 °C for 1 h resulted in a substantial decrease in the hemicellulose and starch content of PPW [46]. The composition of the resulting PPW acid hydrolysate (1% H_2_SO_4_) consisted of fermentable sugars, mainly glucose (35.8 ± 1.25 g/L), with a minor quantity of xylose (2.45 ± 0.25 g/L) and arabinose (0.85 ± 0.05 g/L), and microbial growth inhibitors, mainly furfural (2.1 ± 0.2 g/L) and HMF (1.15 ± 0.08 g/L). After each pretreatment, the obtained PPW hydrolysates were collected and used for PHA production studies. A rise in the H_2_SO_4_ concentration initially showed a positive effect on soluble sugar production; however, beyond a specific level, no significant change in soluble sugar production during pretreatment was recorded [45,47,48].

### 3.4. Physicochemical Changes in PPW After Acid Pretreatment

**Figure 3:**
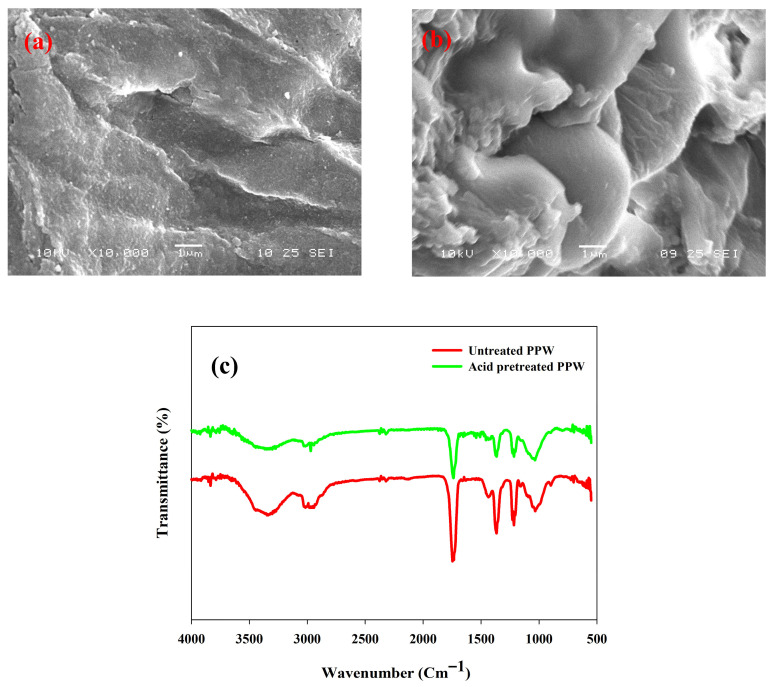
SEM micrographs of (**a**) untreated PPW, (**b**) acid-pretreated PPW, (**c**) FTIR analysis of untreated and acid treated potato peel waste (PPW) biomass.

The morphology of untreated and 1% H_2_SO_4_-pretreated PPW was checked using SEM. SEM images revealed that, after acid pretreatment, noticeable morphological changes in PPW biomass were observed. The homogeneous surface of the biomass became rough and porous, which meant that there was significant depolymerization of the biomass by eradicating lignin and hemicellulose (Figure 3a,b). The structural modifications contributed to a rise in the surface area and enzymatic accessibility, resulting in an enhanced saccharification yield. Other acid-pretreated biomasses have shown similar microstructural changes [49,50].

FTIR analysis was investigated in the range of 4000–400 cm^−1^ to examine relative conformational changes in functional groups during the acid treatment process. The results showed that there was a reduction in the intensity of the carbonyl (C=O) absorption peak at 1740 cm^−1^, which was linked with the side chains of hemicellulose or lignin (Figure 3c). This loss signifies the separation of lignin and the rupture of its connection to hemicellulose because of the impairment of hemicellulose ester bonds during the pretreatment [51].

The wide absorption bands in the 3100–3300 cm^−1^ area are attributed to O-H stretching vibrations, which indicate the breaking of hydrogen bonds of cellulose [52]. In addition, the apparent decline in the absorption at 1035 cm^−1^ (C-O and C=O stretching) and 1385 cm^−1^ (C-H deformation) point to the degradation of the hemicellulose component (Figure 3c) [45]. All these spectral variations serve as evidence of partial dissociation of the molecular interlinking between lignocellulosic components lignin and hemicellulose [50]. FTIR analysis proves that the acid pretreatment enhanced the cellulose content of PPW and was able to remove hemicellulose and lignin, which is supported by compositional data. These structural changes are useful for improved enzymatic hydrolysis and fermentable sugar recovery.

### 3.5. PHA Production Using PPW Acid Hydrolysates by R. eutropha

**Figure 4:**
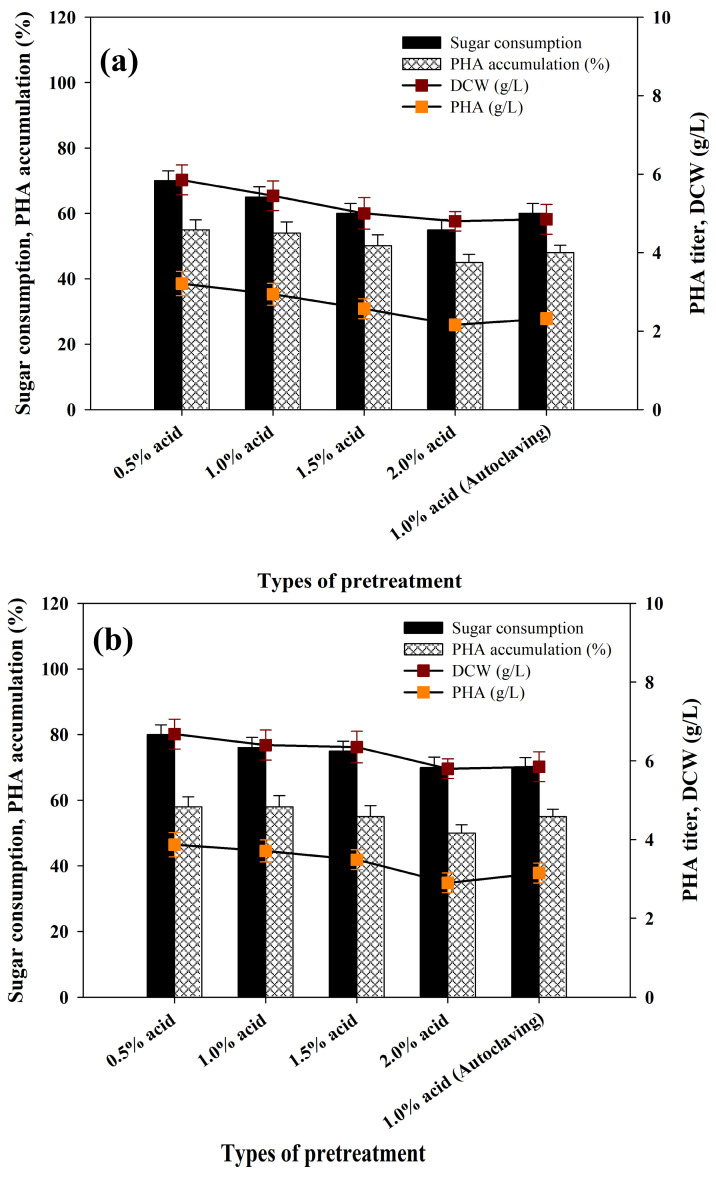
Consumption of sugar, bacteria growth and PHA synthesis parameters by *Ralstonia eutropha* using PPW acid hydrolysates (**a**) before and (**b**) after detoxification of hydrolysates with activated charcoal.

According to the principles of a circular economy, the most rational exploitation of food waste (FW) as a biorefinery feedstock is the solutions of waste throughput, recovery of resources and sustainable production of bio-based products. The approach transforms FW from an environmental liability to an asset, which can be used to accomplish sustainable development and closed-loop bioeconomy goals in the world. Waste feedstocks not only minimize environmental issues that are associated with the disposal of waste but also make the PHA-producing process more sustainable and economically viable. Figure 4a provides a summary of biomass productivity, PHA accumulation and PHA titer by *R. eutropha* using PPW liquid streams (acid hydrolysates) obtained following acid pretreatment at different concentrations. The best findings were a DCW of 5.45 ± 0.40 g/L, PHA accumulation of 54.0 ± 2.15% and a PHB titer of 2.94 ± 0.19 g/L achieved at the end of the 48 h fermentation using 1% H_2_SO_4_-treated PPW hydrolysates (Figure 4a). Acid hydrolysates that were formed in the presence of a higher acid concentration (2% H_2_SO_4_) and physico-chemically pretreated acid hydrolysates showed lower sugar consumption and PHA accumulation by *R. eutropha*. This reduction may be attributed to the availability of inhibitory substances, such as weak acids, furan derivatives and phenolics, that were released during the dissolution of lignin and hemicellulose and adversely affected the PHA fermentation process [47]. To resolve this, the detoxification of PPW acid hydrolysates and further research were conducted to enhance PHA production.

### 3.6. Activated Charcoal Treatment of Acid Hydrolysates

In the acid pretreatment, PPW biomass is broken down, releasing starch, lignin and hemicellulose into a liquid-soluble side stream called PPW acid hydrolysates. This stream is rich in monosaccharides (glucose and xylose), derivatives of organic acids and furanic acids, e.g., 5-HMF and furfural. In accordance with other works, the occurrence of these organic acids and furanic compounds makes it possible to affirm that hemicellulose and lignin were degraded during the process of acid hydrolysis of PPW [53,54]. According to the fermentation profiles, *R. eutropha* showed lower biomass productivity and usage of acid hydrolysates, i.e., sugar usage was in the range of 55 to 68%, which is extremely low (Figure 4a). As a result, the PHA accumulation and PHA yield were unsatisfactory. The low activity of *R. eutropha* in PHA production with PPW acid hydrolysates is explained by the formation of inhibitory by-products during dilute sulfuric acid pretreatment. Major inhibitory compounds that are obtained during pentose and hexose degradation include 2-furaldehyde (furfural), as well as 5-hydroxymethylfurfural (5-HMF), which can significantly inhibit the growth of most microbial species at low concentrations (<0.1 g/L) [55]. Thus, it is necessary to detoxify the hydrolysate [53,56]. A good detoxification strategy must reduce sugar loss, energy consumption, fermentation inhibitors and waste generation.

Activated charcoal (AC) is a low-cost and efficient adsorbent that is highly useful in the removal of metals, dissolved organics and pigment without exhibiting a considerable amount of loss of sugar. This renders it a better option in comparison to ion-exchange resins for the treatment of carbon sources obtained through agricultural wastes [57]. Accordingly, AC detoxification of PPW acid hydrolysates satisfies all requisite pretreatment requirements. AC treatment effectively minimized the inhibitory by-products 5-HMF (60 to 85%) and furfural (75 to 90%) with loss of sugar at only 5.5 to 8.0%; it retained a sugar content of about 89 to 94.5% of the original sugar mass. In order to assess the effects of AC treatment on the growth and PHA synthesis of *R. eutropha,* flask-scale experiments were performed using AC-treated PPW acid hydrolysates. The findings are illustrated in Figure 4b, showing a rise in biomass productivity, PHA accumulation and PHB titer. These values were significantly greater than those achieved in untreated PPW hydrolysates in terms of sugar utilization (76.0 ± 3.15), biomass production (6.40 ± 0.40) and PHA accumulation (58.0 ± 3.4) following AC detoxification (Figure 4a,b).

Similar results were achieved by Jo et al. [58] when recombinant *R. eutropha* NCIMB11599 pKM212-SacC showed a significant enhancement of PHB accumulation (54.1%) and yield (3 g/L) when fermented on activated charcoal-treated sugarcane molasses, though the values were negligible with untreated molasses (0.1%, accumulation and 0.003 g/L yield). In another study, after acid pretreatment, Spruce sawdust hydrolysates detoxified with the help of activated charcoal showed an increase in the PHB yield of 1.45 g/L by *Burkholderia cepacia* [59]. Nevertheless, the growth in the bacterial sugar consumption rate and PHA production were not good enough; therefore, further research is necessary.

### 3.7. Enzymatic Hydrolysis of Acid-Pretreated PPW

Enzymatic saccharification can be considered as an effective hydrolysis approach, mainly because it yields more reducing sugars with the least inhibitors formed [43,60]. In the experiment, acid-pretreated PPW biomass was subjected to enzyme hydrolysis with the aid of a crude enzyme cocktail produced by *Streptomyces* sp. MDS grown on wheat straw under solid-state fermentation in optimized conditions. Utilization of our own produced enzymes could improve the economic performance of the process. The optimization of the enzymatic hydrolysis parameters must be performed in advance to avoid over-utilization of cellulase and to obtain optimal levels of saccharification. It was found that substantial hydrolysis of acid-treated PPW biomass occurred at a temperature of 50 °C and an initial buffer pH of 5.0, using 10 g/L PPW and 30 FPU/g PPW. These conditions were then taken as the most appropriate parameters to use in further experiments. The synergistic effect of the cocktail of enzymes showed a positive effect on biomass depolymerization. The untreated sample of PPW gave a low sugar yield of 70.5 mg/g of PPW which improved in a significant manner after acid pretreatment. Optimizing the H_2_SO_4_ concentration positively influenced the enzymatic yield of PPW to the highest level (425 mg/g of PPW), although there was a marginal decrease in the yield as the acid concentration increased (Table 3). The highest saccharification yield (425 mg/g of PPW), an equivalent hydrolysis yield of 86.4% and a glucose yield of 92.5% were obtained with acid pretreatment with 1% H_2_SO_4_ (Table 3). The intense increase in the sugar yield was attributed to the reduction in the amount of hemicellulose and the alteration in the structure that took place during acid pretreatment (Figure 3a,b). These results affirm that 1% H_2_SO_4_ pretreatment is effective in hydrolyzing PPW biomass, in which the remaining biomass components, mainly cellulose, become more accessible for enzymatic hydrolysis. The saccharification yield, 86.4%, was significantly greater than the level of 30.7 for sulfuric acid-pretreated sugarcane bagasse, as described by Yu and Stahl [55]. Moreover, the amount of reducing sugars formed with 425 mg/g of 1% H_2_SO_4_-treated PPW using a produced cocktail of enzymes by *Streptomyces* sp. MDS was found to be higher than in pine biomass hydrolysis with the use of the enzyme extracts of *Fomitopsis pinicola* (70.9 mg/g) and *Laetiporus sulphureus* (370 mg/g) [61] and sunflower hull hydrolysis with the help of crude enzyme extract of *T. reesei* RUT-C30 (288 mg/g) [62].

### 3.8. PHA Production with Enzymatic Hydrolysates of PPW by R. eutropha

According to a report, *Cupriavidus necator* (previously *Ralstonia eutropha*) has been reported to accumulate PHB to up to 74% of its dry cell weight. PHAs can be potentially used in commercial food packaging, biomedical materials and agricultural products. However, the fact is that half of the total production cost can be linked to the carbon source. Application of PPW as a potential alternative substrate in the production of PHAs can be viewed as an economically efficient and environmentally sustainable solution. Such a practice does not only facilitate the valorization of waste but also assists in fulfilling the growing demand for bioplastics.

**Figure 5:**
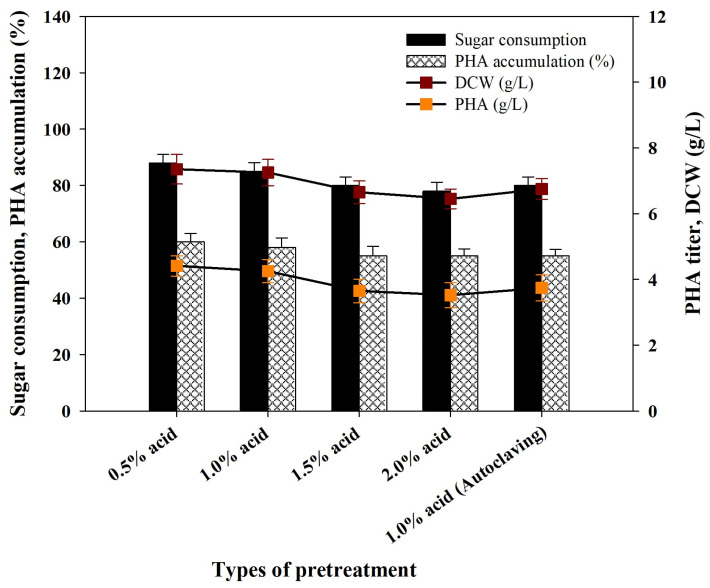
Consumption of sugar, bacteria growth and PHA synthesis parameters by *Ralstonia eutropha* using acid-treated PPW biomass enzyme hydrolysates.

The suitability of acid-pretreated PPW biomass enzyme hydrolysates was assessed in the production of PHA. The pretreatment stage eliminates the majority of inhibitors, including polyphenol derivatives and furan derivatives, with a biomass that is mostly cellulose, then starch and then hemicellulose. As a result, enzyme hydrolysates contain elevated levels of glucose, with minor amounts of xylose and arabinose. *R. eutropha* can efficiently use hydrolysates, although elevated acid concentrations during pretreatment decrease sugar uptake. In ideal conditions, using 1% H_2_SO_4_ PPW enzymatic hydrolysates, a dry cell weight (DCW) of 7.25 ± 0.40 g/L, PHA content of 58.0 ± 3.4% and PHB titer of 4.21 ± 0.28 g/L were attained at the end of 48 hours’ fermentation. Recently, Kag and others used acid-pretreated potato peel waste hydrolysates to produce PHA with *Pseudomonas putida* MTCC 2475 and *Bacillus circulans* MTCC 8167, resulting in PHA accumulations of 28.7 and 29% and PHA yields of 0.60 g/L and 0.232 g/L, respectively [6]. In the recent literature it was stated that *Cupriavidus* sp. KKU38, when cultured on cassava starch hydrolysates, accumulated PHA 61.60% and yielded 2.43 g/L [63]. Cassava pulp was hydrolyzed using 2.5% (*v*/*v*) H_2_SO_4_ under microwave irradiation for PHB production by *Cupriavidus necator* strain A-04. The hydrolysate-to-nutrient ratio of 30:70 (*v*/*v*) gave a cell dry weight of 7.5 ± 0.1 g/L with a PHB content of 66.8 ± 0.3% (*w*/*w*) [64]. In contrast, *Halomonas halophila* with acid pretreatment of cheese whey hydrolysates generated a PHB yield of approximately 3.26 g/L [65] (Figure 5).

### 3.9. Nutrient Supplementation and Osmotic Stress Effects on the Enhancement of PHA Production

**Figure 6:**
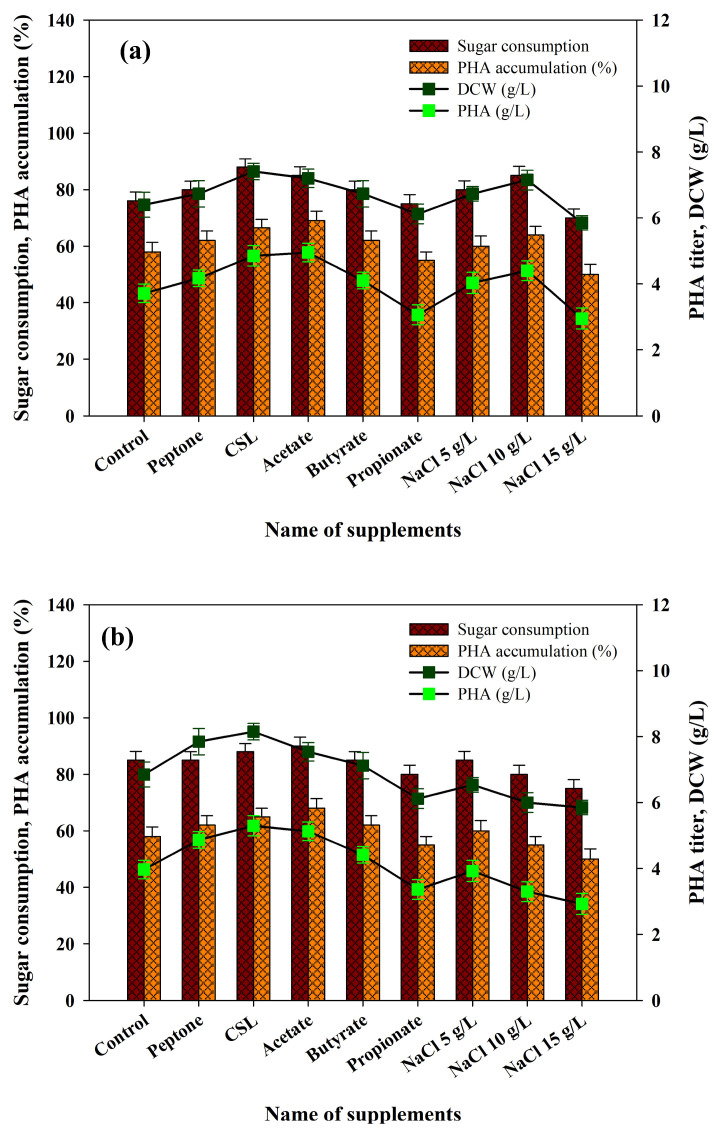
Effects of supplying various nutrient supplements and stress conditions with (**a**) PPW acid hydrolysates and (**b**) PPW enzymatic hydrolysates on *R. eutropha* growth and PHA production.

*Ralstonia eutropha* is one of the most studied microorganisms both in their ability to metabolize a wide spectrum of waste materials and the quantity of PHA produced. Nitrogen can significantly contribute to the growth of PHA-producing microbes and PHA production is sensitive to the availability of nitrogen; thus, nitrogen needs to be carefully controlled at both the stages of growth and production [66]. Nitrogen aids in early cell growth and protein production followed by the inclusion of PHA [67]. Phosphorus plays a significant role as well because it is involved in the formation of cell structures, metabolism and other biochemical processes. Therefore, it is especially important to maintain the adequate concentrations of nitrogen and phosphorus in the medium of production to increase the synthesis of PHA. Earlier, we compared different organic and inorganic sources of nitrogen to enhance the growth of bacteria and the synthesis of PHA [23,24]. In the current study, inoculation was performed in fermentation media in both acid and enzymatic PPW hydrolysates with peptone and corn-steep liquor (CSL). Supplementation of these nitrogen sources in both hydrolysates was effective, with a significant increase in bacterial biomass (7.41 and 7.75 g/L) and PHA accumulation (66.5% and 67.0%) (Figure 6a,b). Other studies have also made similar observations. As an illustration, the monoculture *Bacillus* sp. on agro-residue hydrolysates, especially those supplemented with pretreated sugarcane bagasse by addition of peptone and ammonium nitrate, exhibited increased PHA metabolism. Similarly, *Pseudomonas resinovorans* grown on Cerbera odollam oil with urea as the nitrogen corroborated enhanced PHA production [68]. Chen et al. [69] also showed that addition of 10 g/L of yeast extract into a fermentation medium of recombinant *E. coli* led to dramatic increases in both cell growth (3.02 g/L CDW) and PHB production (1.27g/L P3HB). They suggested that the disaggregation of complex nitrogen sources generates multiple amino acids, thereby lowering the requirement of de novo synthesis of amino acids and thus saving on NADPH. This raises the levels of intracellular NADPH and the ratio between NADPH/NADP^+^ and, eventually, improves the measurement of biomass and PHB [69,70]. These observations support our results.

Additionally, the enrichment of each volatile fatty acid (VFA) which is normally found in acidogenic effluents was compared, establishing their impact on bacterial growth and PHA production. Acetate, butyrate and propionate were chosen to be evaluated in this study. According to the results, acetate supplementation caused significant increases in bacterial growth and PHA accumulation (Figure 6a,b). By contrast, butyrate did not cause any apparent change in bacterial growth in both hydrolysates but caused a 10.5% increase in PHA production in the acid hydrolysates of PPW only (Figure 6a,b). In both hydrolysates, propionate did not produce any significant effect on bacterial growth or on the production of PHA (Figure 6a,b). Acetate is a major precursor of the unit of the HB, so it is likely that it played a role in improvement of growth and worsening of PHA production. Further explanations of the effects of VFAs concerning bacterial growth and PHA accumulation are provided elsewhere [71].

A number of studies have shown that external stress (temperature, exposure to organic solvents (e.g., hydrogen peroxide and alcohol), UV radiation or osmotic stress) can stimulate PHA production. Passanha et al. [72] explored the effects of various levels of NaCl on PHA production of *Cupriavidus necator*. They demonstrated that addition of the production medium with 9 g/L NaCl increased PHA production by about 30%. It was suggested that PHA accumulation protects bacteria against osmotic fluctuations. Further, PHA accumulation in different microbial cultures has been proven to be induced by mild osmotic stress. Indicatively, Obruca et al. [73] found that regulated osmotic pressure is a significant factor in maximizing PHA production in *C. necator.* The impact of three NaCl concentrations (5, 10 and 15 g/L) on PHA production in cultivated *R. eutropha* using acid and enzymatic PPW hydrolysates were evaluated in the current study. The findings proved that NaCl supplementation at up to 10 g/L stimulated bacterial development and PHA production within both hydrolysates (Figure 6a,b). Nevertheless, growth of biomass and PHA accumulation decreased by 15g/L NaCl. These results indicate that productivity of PHA can be improved by ensuring optimal osmotic pressure in the production medium. Therefore, NaCl addition is a simple, low-cost, sustainable, non-toxic and non-reactive extrinsic stress measure worth adopting to enhance PHA production, where large-scale applications are to be considered. Under optimal conditions, the production media containing acid and enzymatic hydrolysates of PPW with the supplementation of CSL showed the maximum bacterial cell biomass (7.41 and 7.75 g/L), PHA accumulation (66 and 67%) and PHA titer (4.85 and 5.19 g/L), respectively. The obtained results were found to be comparable with the previous literature on food waste biomass (Table 4).

### 3.10. PHA Characterization

#### 3.10.1. XRD Analysis

**Figure 7:**
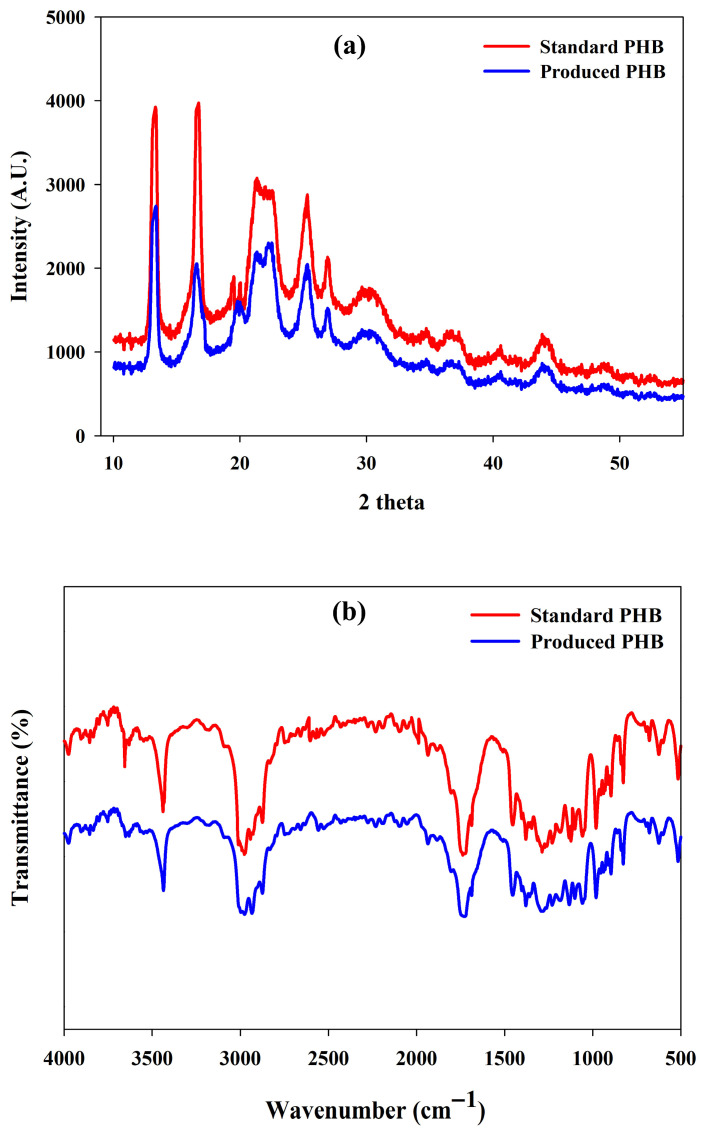
(**a**) XRD diffractogram pattern and (**b**) FTIR spectral analysis of the produced PHA by *R. eutropha* using PPW hydrolysates.

The X-ray diffraction (XRD) pattern of the PHB produced clearly showed that the material was crystalline in nature. The definite and sharp peaks of diffraction at the 2θ values of 13.34° and 16.38° indicated the existence of crystalline regions in the polymer matrix. The other reflections at 21.08° and 22.51° confirmed the presence of orthorhombic crystal structures, which is also typical of α-form PHB crystals (Figure 7a). In addition, there were some small peaks that could be observed at 25.33° and 27.13°, which suggest the existence of partially crystalline parts, which are well-organized but still heterogeneous crystalline structures. The higher crystallinity of PHA is advantageous in terms of improved mechanical strength, higher thermal stability, better permeability properties and slower biodegradation. These attributes enhance the suitability of PHA in food packaging and biomedical applications. The diffraction profile overall is consistent with the XRD patterns reported previously of PHB, confirming the crystallinity of the polymer produced in this work [66,82].

#### 3.10.2. FTIR Analysis

Fourier Transform Infrared (FTIR) spectroscopy was used to determine the molecular structure and functional group composition of extracted PHA. The wide absorbance at 3440 cm^−1^ matched the stretching vibration of terminal hydroxyl (-OH) groups, and the C-H stretching vibrations of the aliphatic chains were near 2974 cm^−1^. The presence of ester carbonyl (C=O) functional groups of PHB was confirmed by a strong and clear peak at 1720 cm^−1^ (Figure 7b).

Further peaks at 1470 cm^−1^ and 1380 cm^−1^ were the asymmetric and symmetric bending vibrations of the –CH_2_ and –CH_3_ groups, respectively (Figure 7b). The band at 1280 cm^−1^ was attributed to the rotational motion of -CH groups, and the absorption at 1045 cm^−1^ indicated C-O-C stretching observed with ester bonds. Also, several absorption characteristics in the area of 978–513 cm^−1^ revealed C–O and C–O–C modes of absorption, and it further indicated the presence of a polymer ester structure (Figure 7b). These typical peaks are in line with the spectral data described in the literature, hence validating that the product obtained was poly(3-hydroxybutyrate) (PHB) [23,82,83].

#### 3.10.3. TGA and DSC Analysis

**Figure 8:**
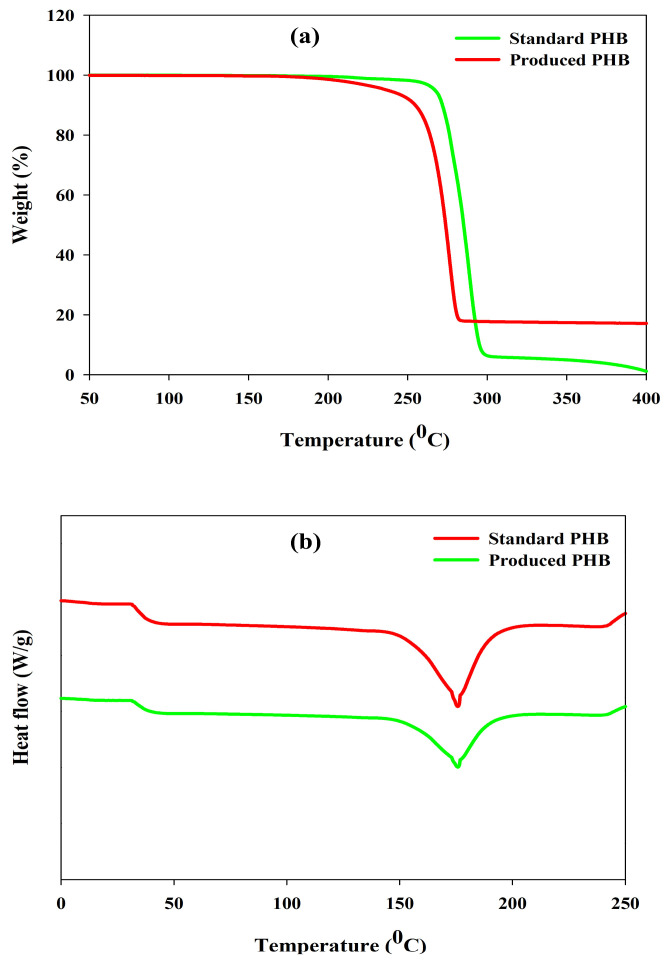
(**a**) TGA and (**b**) DSC analysis for the evaluation of thermal characteristics of produced PHA by *R. eutropha* using PPW hydrolysates.

Thermogravimetric (TGA) and differential scanning calorimetric (DSC) analyses were performed to determine the thermal stability of the produced PHB and its melting properties, as these characteristics are significant factors that need to be considered in relation to its potential industrial use and storage conditions. The TGA thermogram (Figure 8a) demonstrated a one-step degradation rate profile and upon examination; the decomposition commenced at 291 °C which indicates high thermal resistance. The loss of mass observed is associated with cleavage of ester linkages by a *β*-elimination reaction. The DSC thermogram (Figure 8b) showed a melting temperature (*Tm*) of 175.4 °C, which is very close to the standard PHB temperature (178 °C). The thermal and structural analyses together indicate that the produced PHB was a crystalline and thermally stable biopolymer and was in line with the physicochemical characteristics of high-purity PHB in the literature [6,84,85].

## 4. Conclusions

An integrated biorefinery to produce bioextractives and PHA using PPW as the starting material can provide a sustainable and economically feasible solution. In this work, we have generated a multi-step conversion plan to reach complete valorization of PPW: (i) bioactive compounds with antioxidant and antidiabetic properties were extracted, (ii) extracted biomass was subjected to acid hydrolysis, producing a liquid stream to be converted into PHA and (iii) the leftover biomass was further hydrolyzed using an in-house enzyme cocktail and utilized to generate PHA. This strategy allows for effective recycling of PPW within an integrated biorefinery to make several value-added products. Under optimized fermentation conditions, *R. eutropha* with PPW acid and enzyme hydrolysates produced a maximum PHA accumulation of 66% and 67% and PHA titers of 4.85 g/L and 5.19 g/L, respectively. The structural and thermal properties of produced PHB were found to be similar to those of standard PHB. In our lab the production of enzymes is already at an established stage; more experiments and up-scaled investigations should be conducted to increase the economic viability of the process. Despite these promising results, to achieve large-scale, environmentally friendly production of PHB with the use of PPW, process optimization, bio-reactor configuration and a complete techno-economic evaluation will be necessary to evaluate actual commercial feasibility. Finally, this paper creates a sustainable system of PPW-to-PHA conversion, promoting the aims of a sustainable bioeconomy, and can be extended to other forms of food waste.

## Figures and Tables

**Table 1:** Determination of extract yield and total phenolic and total flavonoid contents of potato peel waste after methanol extraction.

Type of Extraction	Solvent Used	Extract Yield (%)	Total Phenolics (mg GAE/g DW)	Total Flavonoids (mg QE/g DW)
Solvent extraction	Methanol	10.58 ± 0.85	2.61 ± 0.45	0.806 ± 0.05

Values are the mean of three experiments; (±) standard error; (SE) by one-way ANOVA with Tukey–Kramer multiple comparisons test.

**Table 2:** Chemical composition of solvent extracted potato peel waste (PPW).

Name of Component	Chemical Composition (%)
Cellulose	20.82 ± 0.78
Hemicellulose	7.02 ± 0.54
Lignin	11.29 ± 0.85
Starch	38.81 ± 1.54
Proteins	10.45 ± 1.15
Fats/Lipids	1.45 ± 0.05
Ashes	7.55 ± 0.48

Values are the mean of three experiments; (±) standard error; (SE) by one-way ANOVA with Tukey–Kramer multiple comparisons test.

**Table 3:** Effect of applied pretreatment to potato peel waste on biomass recovery, lignin removal, hydrolysis yield and glucose yield after enzymatic hydrolysis (crude enzyme dosage 30 FPU/g of PPW).

Pretreatment Conditions	Material Recovery (%)	Lignin Removal (%)	TRS (mg/g of PPW)	Hydrolysis Yield (%)	Glucose Yield (%)
Untreated	100 ± 0.0	ND	70.5 ± 2.25	9.5 ± 0.48	80.0 ± 2.35
Thermal (100 °C, 3 h)	78.5 ± 2.15	30.8 ± 1.25	210.0 ± 5.85	35.5 ± 1.25	82.5 ± 3.14
0.5% H_2_SO_4_ (100 °C, 3 h)	64.5 ± 1.85	50.8 ± 2.15	316.8 ± 6.25	54.6 ± 2.18	88.0 ± 3.45
1.0% H_2_SO_4_ (100 °C, 3 h)	52.5 ± 2.05	72.5 ± 3.56	425.0 ± 6.45	86.4 ± 2.45	92.5 ± 4.15
1.5% H_2_SO_4_ (100 °C, 3 h)	48.5 ± 1.98	80.0 ± 4.25	400.5 ± 5.85	88.8 ± 3.45	94.1 ± 4.25
2.0% H_2_SO_4_ (100 °C, 3 h)	42.5 ± 1.65	88.3 ± 4.98	380.4 ± 4.98	87.5 ± 3.85	94.0 ± 4.40
1.0% H_2_SO_4_ (Autoclaving 121 °C, 20 min)	55.5 ± 1.85	65.5 ± 2.54	356.5 ± 4.65	71.3 ± 2.92	90.5 ± 4.18

ND = not determined; PPW: potato peel waste. Values are the mean of three experiments; (±) standard error; (SE) by one-way ANOVA with Tukey–Kramer multiple comparisons test.

**Table 4:** Comparison of growth parameters, PHA accumulation and PHB production using lignocellulosic biomass hydrolysates by *Ralstonia eutropha* strains.

Name of Food Waste Materials	Name of Strain	Operation Mode	Fermentation Period (h)	DCW (g/L)	PHA (%)	PHA Titer (g/L)	Type of PHA	References
Kitchen waste	*C. necator* CCGUG 52238	Batch	20	4.6	52.79	2.42	PHB	[74]
Waste frying rapeseed oil	*C. necator* H16	Flask, batch	40	10.8	67.9	7.33	PHB	[75]
Waste frying palm oil				11.9	58.0	6.90		
Waste frying sunflower oil				12.53	52.4	6.56		
Vinasse and enzyme-digested sugarcane molasses	*C. necator* DSM 545	Batch	48	20.8	56.0	11.7	PHB	[76]
Apple pulp waste	Co-culture of *C. necator* and *Pseudomonas citronellolis*	Shake flasks	48	5.51	33.6	1.85	PHB (48%) and mcl-PHA (52%)	[77]
Grape winery waste	C. *necator*	Shake flasks	72	4.1	47.2	1.9	PHB	[78]
Soluble potato starch	*C. necator* DSM 545 (recombinant)	Batch	96	5.70	61.6	3.51	PHB	[79]
Broken rice				13.29	43.3	5.78		
Purple sweet potato waste				10.21	36.0	3.65		
Raw corn starch				12.28	48.2	5.92		
H_3_PO_4_-treated pineapple peel hydrolysate	*C. necator* strain A-04	Batch	72	6.1	35.6	2.1	PHB	[80]
H_2_SO_4_-treated pineapple core hydrolysate			84	5.3	12.7	0.7		
Makgeolli Lees Enzymatic Hydrolysate with glucose	*R. eutropha* H-16	Fed Batch	72	36.9	79.3	24.1	PHB	[81]
Potato peel waste acid hydrolysates	*R. eutropha* ATCC 17699	Batch	48	7.41	66	4.85	PHB	This study
Potato peel waste enzymatic hydrolysates				7.75	67	5.1		

## Data Availability

The raw data supporting the conclusions of this article will be made available by the authors on request.
